# Bidirectional interplay between SARS-CoV-2 and autophagy

**DOI:** 10.1128/mbio.01020-23

**Published:** 2023-07-12

**Authors:** Hao Zhou, Zhiqiang Hu, Sergio Castro-Gonzalez

**Affiliations:** 1 Department of Microbiology and Immunology, College of Medical Technology, Chengdu University of Traditional Chinese Medicine, Chengdu, China; 2 Shandong New Hope Liuhe Agriculture and Animal Husbandry Technology Co., Ltd, Dezhou, China; 3 Biochemistry and Molecular Biology I, University of Granada, Granada, Spain; Albert Einstein College of Medicine, Bronx, New York, USA

**Keywords:** autophagy, COVID-19, innate immunity, SARS-CoV-2, therapeutic targets

## Abstract

Severe acute respiratory syndrome coronavirus 2 (SARS-CoV-2), as the causative agent of the recent COVID-19 pandemic, continues representing one of the main health concerns worldwide. Autophagy, in addition to its role in cellular homeostasis and metabolism, plays an important part for the host antiviral immunity. However, viruses including SARS-CoV-2 have evolved diverse mechanisms to not only overcome autophagy’s antiviral pressure but also manipulate its machinery in order to enhance viral replication and propagation. Here, we discuss our current knowledge on the impact that autophagy exerts on SARS-CoV-2 replication, as well as the different counteracting measures that this virus has developed to manipulate autophagy’s complex machinery. Some of the elements regarding this interplay may become future therapeutic targets in the fight against SARS-CoV-2.

## SARS-CoV-2 AND AUTOPHAGY AT A GLANCE

### SARS-CoV-2

The emergence of new virus infections always leads to great public health concerns. The most recent example is the severe acute respiratory syndrome coronavirus 2 (SARS-CoV-2), which rapidly spread worldwide in late 2019 and caused the ongoing COVID-19 pandemic. Human coronaviruses (CoVs) are one of the most devastating virus families that frequently infects humans. CoVs are single-stranded positive-sense RNA viruses divided into α-CoVs (including HCoV-NL63 and HCoV-229E) and β-CoVs (including HCoV-OC43, HCoV-HKU1, SARS-CoV, MERS-CoV, and SARS-CoV-2) (1 - 3).

**Fig 1:**
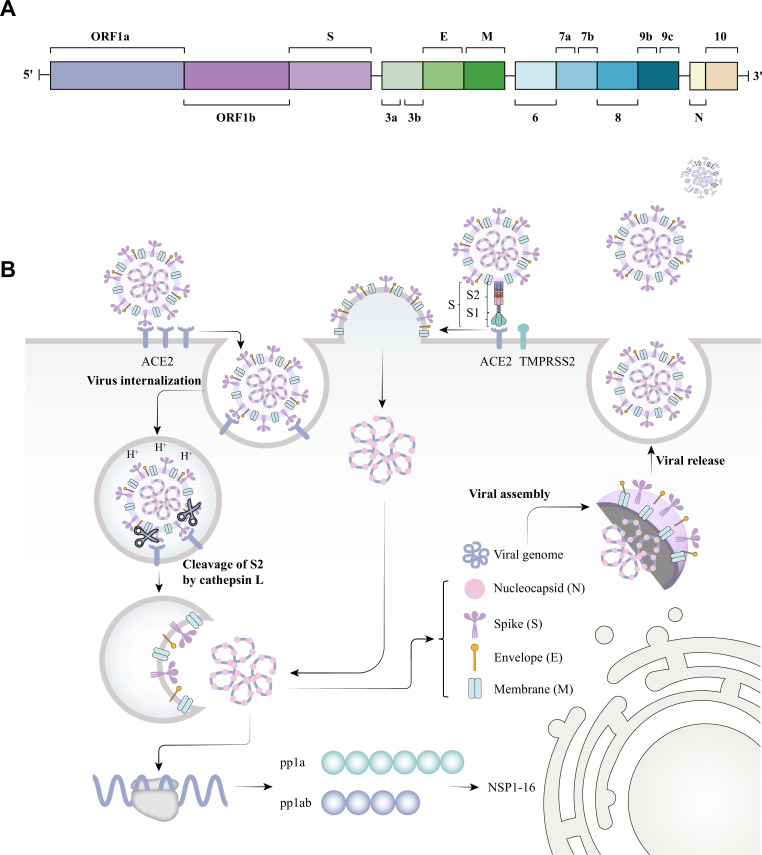
(**A**) SARS-CoV-2 genome encodes ORF1a and ORF1b, which give rise to pp1a and pp1ab, respectively. The polyproteins pp1a and pp1ab generate the non-structural proteins NSP1 to NSP16. SARS-CoV-2 genome also encodes four structural proteins, including a membrane protein (M), an envelope protein (E), a nucleocapsid (N), a spike (S), and a set of accessory proteins (ORF3-10). The spike protein S is cleaved into S1 and S2 subunits at the furin cleavage site, with the S1 subunit acting as a surface binding motif to ACE2 on the cell membrane. The subunit S2 is involved in the subsequent membrane fusion to enable viral entry. (**B**) The replication cycle of SARS-CoV-2 comprises a sequence of events that involves viral binding, the cleave of the S protein by TMPRSS2 or Cathepsin L, membrane fusion, viral RNA release, protein translation and proteolysis, sub-genomic RNA replication and transcription, viral assembly, and the release of mature virions.

COVID-19 is caused by SARS-CoV-2 infection and replication within epithelial cells of the respiratory tract. The entry of SARS-CoV-2 into host epithelial cells is mediated by the direct binding of the viral spike protein (S) and the host cell membrane protein angiotensin-converting enzyme 2 (ACE2), and its subsequent cleavage by the host proteases TMPRSS2 or Cathepsin L. After entering the cell, SARS-CoV-2 synthesizes a set of structural (to be incorporated into newly formed virions) as well as non-structural proteins in order to efficiently generate new viral particles that will later exit the cell through exocytosis to complete its life cycle (Fig. 1) (4 - 6).

SARS-CoV-2 genome encodes a total of 29 proteins. It synthesizes the polyproteins ORF1a (pp1a) and ORF1b (pp1ab), which will generate the non-structural proteins 1–16 (NSP1–16). It also encodes structural proteins that will be part of mature virions, which include the spike (S), envelope (E), membrane (M), and nucleocapsid (N) protein. Additionally, SARS-CoV-2 synthesizes a set of accessory proteins known as open reading frame (ORF) proteins 3–10 (ORF 3–10), including ORF3a, ORF7a, ORF8, or ORF10, whose main function is the enhancement of viral replication using different mechanisms. Some non-structural proteins possess enzymatic activities such as proteases or polymerases; NSP3 (papain-like protease), NSP5 (protease), NSP12 (RNA-dependent RNA polymerase), NSP13 (helicase/triphosphatase), NSP14 (exoribonuclease), NSP15 (endonuclease), and NSP16 (methyltransferase) (6 - 9). SARS-CoV-2 has also evolved some strategies to manipulate the cellular machinery in order to regulate its own replication and exocytosis. A systematical study showed that non-structural proteins such as NSP1, NSP3, NSP5, NSP10, NSP13, NSP14, ORF3a, ORF6, ORF7a, and ORF7b served as primary viral innate immune antagonists (10).

Extended research has been conducted during the past years regarding the interactions between human CoVs and the host innate immunity, with a special emphasis on SARS-CoV-2. Some of these studies aim to better understand the molecular interplay between SARS-CoV-2 and the immune-related process of autophagy. Although much has been achieved in this context, how SARS-CoV-2 infection modulates the cellular machinery, and in particular autophagy, still remains unclear.

### Autophagy

Autophagy is as a “self-eating” process that mediates the degradation and recycling of a wide variety of cellular components through the lysosome. The general term of autophagy encompasses three primary types: microautophagy, chaperone-mediated autophagy (CMA), and macroautophagy. Microautophagy involves the direct engulfment of cytoplasmic material by lysosomes through the invagination of the lysosomal membrane (11). Chaperone-mediated autophagy involves the selective degradation of specific proteins that are recognized by chaperone proteins and delivered to lysosomes through a specific receptor-mediated pathway (12). Finally, macroautophagy involves the formation of double-membrane structures called autophagosomes that engulf cytoplasmic material and deliver it to lysosomes for degradation (13, 14). Macroautophagy is the most widely studied type of autophagy, and it is usually (including our manuscript) regarded as autophagy.

Macroautophagy (hereafter autophagy) is an intracellular degradative axis highly conserved among all eukaryotic organisms. Autophagy plays a crucial housekeeping role in the cell by promoting the lysosomal degradation of misfolded proteins and clearance of damaged or dysfunctional organelles, which can become an essential source of energy during nutrient deprivation periods (15 - 17). Additionally, autophagy is also activated in the presence of intracellular pathogens, such as viruses, being able to target them for their direct lysosomal degradation, which in turn, also enhances secondary immune processes against those pathogens (18 - 20). Specifically, the peptides generated by the autophagic degradation of intracellular pathogens are exposed at the surface of infected cells by MHC-I and MHC-II complexes to be recognized by immune cells, enabling further immune responses to control the infection (21 - 24).

Autophagy mediates the delivery of its cargo to lysosomes through very specialized double-membrane vesicles known as autophagosomes, in a tightly regulated process that involves different subsequent steps and more than 30 autophagy-related proteins (ATGs) (17, 25).

## THE AUTOPHAGY MACHINERY AND REGULATION

### Autophagy initiation

There are several autophagy-initiation signals reported, and the mechanistic target of rapamycin kinase complex 1 (mTORC1), the main autophagy inhibitor, is the convergence of most of these signals, being regarded as a key switch of autophagy (26, 27). mTORC1 is found constitutively active, however, under stress situations, such as starvation or infection by intracellular pathogens; mTORC1 is suppressed by upstream signals, which lifts in turn, the inhibitory effect that mTOR1 exerts over the autophagy pathway (28). Three major pathways are known to regulate the activity of mTORC1: (i) PI3K/AKT/mTORC1, (ii) RAS/RAF/MEK/ERK/mTORC1, and (iii) AMPK/mTORC1 pathways.

#### PI3K/AKT/mTORC1

This axis involves the Class I phosphatidylinositol 3-kinase (PIK3K/PI3K) and AKT, and it is regulated by a variety of cell growth and survival signals (29 - 31).

#### RAS/RAF/MEK/ERK/mTORC1

This pathway involves the sequential activation of rat sarcoma (RAS), rapidly accelerated fibrosarcoma (RAF), mitogen-extracellular activated protein kinase kinase (MEK), and the extracellular-signal-regulated kinase (ERK) (32, 33). Different stimuli, such as the presence of growth factors, hormones, cytokines, and environmental stress, could lead to the activation of this pathway (34).

#### AMPK/mTORC1

Adenosine monophosphate-activated protein kinase (AMPK) is an energy-sensing kinase that in energy shortage conditions can inhibit the activity of mTOR to, among other things, start the autophagic process (16, 28, 35). In addition, AMPK can also activate the uncoordinated 51-like kinase 1 (ULK1) and the Class III PI3K complex, which act downstream of mTORC1 and regulate the initiation of autophagy (36, 37).

**Fig 2:**
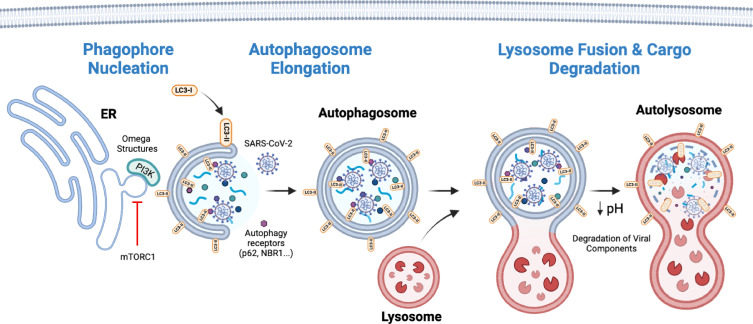
Representation of the key players and main stages of the autophagy pathway, including phagophore formation, autophagosome elongation, lysosomal acidification, and fusion as well as cargo degradation. Additionally, the figure depicts the antiviral effect of autophagy through the recruitment of viral components for their ultimate autolysosome-mediated degradation.

As mentioned above, the canonical initiation of autophagy requires the inhibition of mTORC1, which in normal conditions phosphorylates and represses the activity of the autophagy initiator ULK1. The kinase ULK1 is the catalytic subunit of the so-called autophagy initiation complex that also contains the scaffold proteins ATG13, ATG101, and focal adhesion kinase family interacting protein of 200 KD (FIP200) (25, 38). ULK1 initiation complex is able to mediate the phosphorylation and activation of the Class III PI3K. The Class III PI3K complex contains Beclin 1 (BECN1), ATG14, phosphoinositide-3-kinase regulatory subunit 4 (PIK3R4/VPS15), and the phosphatidylinositol 3-kinase catalytic subunit type 3 (PIK3C3/VPS34) (38 - 41). The kinase activity of VPS34 then generates phosphatidylinositol 3-phosphate (PI3P) on different target membranes, such as the endoplasmic reticulum (ER), that will recruit the last effectors involved in the initiation of autophagy: WD repeat domain, phosphoinositide interacting 2 (WIPI2), and the zinc finger FYVE-type containing 1 (ZFYVE1/DFCP1) (42, 43). WIPI2 and DFCP1 bind to membranes through PI3P, where they facilitate the formation of omega structures (Ω) at the ER surface (phagophore nucleation) and recruit downstream ATGs that will mediate the subsequent elongation of autophagosomes at those places (Fig. 2) (43, 44). Interestingly, in addition to the above-described canonical initiation of autophagy, immune effectors such as the cyclic GMP-AMP synthase (cGAS) and the stimulator of interferon response cGAMP interactor 1 (STING1) are known to induce a non-canonical (ULK1- and BECN1-independent) autophagic process as a defense mechanism in response to viral infections (45, 46).

### Autophagosome elongation

Membrane-bound WIPI2 mediates the recruitment of the ATG12-ATG5-ATG16L1 complex to phagophores assembly sites (nascent autophagosomes) through the direct interaction with the subunit ATG16L1. ATG12-ATG5-ATG16L1 complex is essential for the elongation of autophagosomes, and its formation requires a series of ubiquitin-like reactions mediated by ATG7 (E1) and ATG10 (E2) (47 - 49). The function of the ATG12-ATG5-ATG16L1 complex is one of the most characteristics of this pathway, mediating the lipidation and thus, incorporation of the microtubule-associated protein 1 light chain 3 beta (MAP1LC3B/LC3) to both the inner and the outer membrane of autophagosomes (39). The modification of LC3 is another ubiquitin-like reaction in the process of autophagosome formation that requires ATG7 (E1), ATG3 (E2), and ATG5-ATG12-ATG16L1 (E3) (48). LC3 is synthesized as pro-LC3, then ATG4 cleaves its C-terminus, turning pro-LC3 into LC3-I. When autophagy is activated, LC3-I is lipidated through the ubiquitin-like reaction that conjugates LC3-I to phosphatidylethanolamine (PE) to forming autophagosome membranes. The lipidated version of LC3, which decorates autophagosome membranes, is known as LC3-II and plays a central role in autophagosome elongation and the recruitment of autophagic cargo (50 - 52). For the latter, the autophagy machinery counts with a set of receptors such as SQSTM1/p62, NBR1, or TOLLIP, among others. These receptors bind simultaneously to specific autophagic cargo (very often found tagged through ubiquitination) on one side, and to LC3-II located on the inner membrane of autophagosomes, through the so-called LC3-interacting region (LIR) (53 - 55). Finally, the autophagosome grows around the cargo and engulfs it, along with the receptors used for its recruitment, prior to its delivery to lysosomes for degradation (Fig. 2).

### Autophagic degradation

The degradation of autophagy cargo mainly depends on the formation of autolysosomes, which are generated after the fusion between mature autophagosomes and lysosomes, or alternatively, autophagosomes, late endosomes, and lysosomes (56 - 58). The fusion process requires the participation of multiple factors associated with vesicle trafficking pathways. For instance, the phosphoinositides PI(3)P and PI(4)P are located on autophagosomes, late endosomes, and lysosomes, and have been reported to be involved in autophagosome-lysosome fusion (59, 60). Additionally, Rab7, a member of the Small GTPase family Rab, is able to recruit various effector proteins to these membranes, including motor proteins and tethering factors, which are required for the subsequent events that lead to the fusion between autophagosomes and lysosomes (61, 62). Finally, multiple tether and adaptor proteins, such as homotypic fusion and vacuole protein sorting complex (HOPS complex) (63, 64), ATG14 (65), and some other factors (66), are also involved in this complex process. Among these factors, we can find the SNARE complex that can be formed by Syntaxin17 (STX17), synaptosome-associated protein 29 (SNAP29), or vesicle-associated membrane proteins 7 and 8 (VAMP7 and VAMP8) (67). STX17 is translocated from the ER to autophagosomes, and the association of STX17 may be facilitated by LC3-II and lysosome-associated membrane protein type 2 (LAMP2) (66, 68). LAMP2, while representing one of the hallmarks of CMA, is also important for the progression of the late stages of macroautophagy (68 - 70). On the other hand, SNARE VAMP7/8 is located on the membranes of late endosomes and lysosomes. SNAP29 acts as the bridge between STX17 and VAMP7/8 to promote the fusion process between lysosomes and autophagosomes, leading to the delivery of the autophagic content into lysosomes for its degradation through the action of the hydrolases present in these organelles (Fig. 2) (65, 71). The lysosomal acidification is a required step for the activation of those hydrolases and thus, the ultimate degradation of the autophagic cargo in autolysosomes (66, 72, 73).

## INTERPLAY BETWEEN AUTOPHAGY AND SARS-COV-2

As it occurs in the context of other viral infections, including closely related viruses (e.g., SARS-CoV or MERS) (74, 75) or from unrelated families (e.g., HIV or West Nile virus) (76 - 78), autophagy seems to have the capacity to act as a defense mechanism to suppress SARS-CoV-2 viral replication by targeting the virus for autolysosome degradation (Fig. 2) (79). However, additional contradictory findings made evident a viral dependency to this pathway. SARS-CoV-2 has developed different strategies to counteract autophagy, or even hijack its machinery for its own replicative purposes. In the following text, we summarize how SARS-CoV-2 viral proteins impact autophagy and vice versa, in an attempt to better understand this complex intertwined relationship.

### Autophagy activation inhibits SARS-CoV-2

As already mentioned, autophagy seems to be triggered in the context of SARS-CoV-2 infection to act as a protective measure. It has been reported that in the presence of SARS-CoV-2, there is an activation of ULK-1-Atg13 and VPS34-VPS15-BECN1 in order to promote autophagosome formation (80). Moreover, the downregulation of the autophagy initiator ATG5, ATG7, BECN1, and FIP200 using siRNAs significantly reduces SARS-CoV-2 replication *in vitro* (81). Additionally, autophagy-related elements, such as the lysosomal protein LAMP2, co-localize with viral proteins and largely impair viral RNA replication by interacting with the 5′ untranslated region (UTR) of SARS-CoV-2 (82). Although most studies focus on the interaction between macroautophagy and SARS-CoV-2, taking into account the central role of LAMP2 in CMA, further studies would be required to clarify the potential implication of CMA in SARS-CoV-2 replication.

In line with those observations, and as further detailed in the following sections, different studies have shown that the pharmacological activation of both, the canonical autophagy and non-canonical autophagy, has an inhibitory effect on SARS-CoV-2 replication and propagation (81, 83, 84). However, despite the antiviral potential of the autophagy machinery, SARS-CoV-2 has evolved a wide variety of strategies to avoid this repression.

### SARS-CoV-2 hijacks autophagy to promote viral replication

Although in the presence of SARS-CoV-2, there is an increase in autophagosome formation, SARS-CoV-2 can prevent the late events of autophagy, and therefore, it causes an incomplete response that seems to be favorable for viral replication (80, 81, 85, 86). In line with this, some studies have shown that SARS-CoV-2 may intentionally activate autophagy to boost its own replication. First of all, SARS-CoV-2 infection leads to the upregulation of ROS, which in turn, suppresses the PI3K/AKT/mTOR pathway and promotes autophagy activation (87). Additionally, SARS-CoV-2 activates the Class III PI3K to generate PI3P, resulting in the recruitment of DFCP1 to phagophores and thus, the formation of double-membrane vesicles (DMVs). DMVs have been suggested to be exploited by SARS-CoV-2 to act as RNA replication organelles (88). To avoid the detrimental effect of autophagy, SARS-CoV-2 uses different mechanisms that ultimately prevent the degradation of the autophagic cargo, and therefore, it leads to the accumulation of markers such as LC3 or p62 in infected cells. This phenotype has been observed *in vitro*, *in vivo* as well as in lung samples from COVID-19 patients (80, 81).

Interestingly, and supporting the hypothesis that SARS-CoV-2 utilizes autophagy for its own replicative benefit, genome-wide CRISPR knockout screenings have identified several autophagy-related genes such as TMEM41B to be common host dependency factors required for the replication of SARS-CoV-2 and other closely related coronaviruses (89, 90).

### How SARS-CoV-2 proteins modulate autophagy

**Fig 3:**
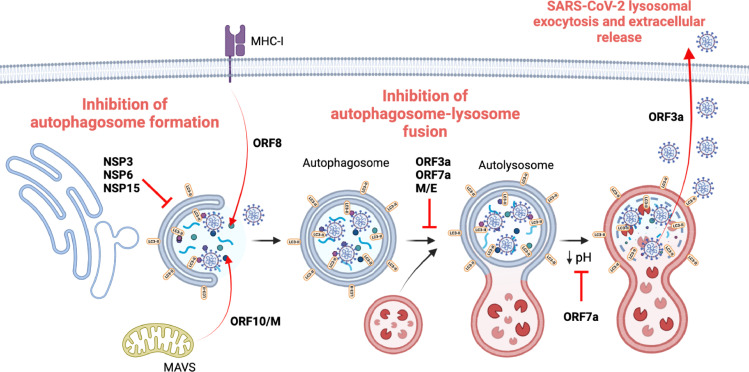
Summary of the different effects of SARS-CoV-2 proteins on autophagy machinery during a productive viral infection. The figure depicts the inhibition of autophagy mediated by SARS-CoV-2 at the early stages (autophagosome formation) and the late stages (lysosome acidification and autolysosome formation). Additionally, SARS-CoV-2 promotes immune evasion by facilitating the delivery of MAVS (via mitophagy) and MHC-I molecules for autophagic degradation.

The number of proteins and approaches that SARS-CoV-2 has evolved in order to harness autophagy further highlights the importance of this interaction for viral fitness (Fig. 3).

SARS-CoV-2 ORF3a interacts with autophagy at different levels. This accessory protein is able to prevent the fusion between autophagosomes and lysosomes, decreasing the autophagic flux and thus, providing an immune escape from autophagy (10, 85, 91). Mechanistically, ORF3a induces an incomplete autophagic process in a FIP200/BECN1-dependent manner; however, it ends preventing autolysosome formation by blocking HOPS-mediated assembly of the SNARE complex (85, 92). Moreover, ORF3a effect on endolysosomal compartments promotes lysosomal exocytosis and enhances extracellular viral release (93). In addition to its ability to modulate the canonical autophagy, SARS-CoV-2 ORF3a has also been reported to be able to counteract the flux and thus, modulating the antiviral effect of the non-canonical STING1-mediated autophagy (94).

ORF7a also dysregulates the late stages of autophagy, but in this case, by inhibiting the acidification of lysosomes (Fig. 3) (10, 95). Additionally, ORF7a can also prevent autophagosome-lysosome fusion, but unlike ORF3a, ORF7a achieves this by promoting the degradation of the SNARE protein SNAP29, which is crucial for autophagosome and lysosome fusion (10, 96). In addition, SARS-CoV-2 can also block autophagy turnover through the structural proteins M and E, which ultimately leads to the accumulation of autophagosomes and p62 in the cell (10, 95). Importantly, the inhibition of the autophagic degradation might be facilitating the translocation and stabilization of M onto autophagosomes that will facilitate virion release (Fig. 3) (97).

SARS-CoV-2 ORF8, far from inhibiting autophagy, is able to selectively target MHC-Ι for lysosomal degradation through autophagy (Fig. 3), which weakens the antiviral immune surveillance (98). Similarly, ORF10 and M are able to counteract innate immunity by promoting the autophagic degradation of MAVS (through mitophagy) (Fig. 3), which are important antiviral elements associated with mitochondria, leading to a reduction in interferon I (IFNI) production (99, 100). Additionally, and sharing the goal with ORF3a, ORF10 can also counteract non-canonical autophagy by inhibiting STING1 activation (101).

Several non-structural proteins also possess the capacity to downregulate autophagy. For instance, the SARS-CoV-2 papain-like protease NSP3 reduces the starvation-induced autophagy and disrupts the formation of the initiation complex that involves ULK1 and ATG13 (102). The viral protein NSP6 is able to inhibit the initiation of autophagy by preventing the formation of pre-autophagosomal structures (103). The helicase NSP13 can mediate the autophagic degradation of TBK1 in a p62-dependent manner, which impairs production of IFNI and therefore, attenuates the host innate immunity (104). Similarly to the effect exerted y NSP6, NSP15 can also hinder early events of autophagy resulting in the reduction of autophagosome formation (Fig. 3) (10).

### Autophagy interacts with the receptor ACE2 to regulate viral internalization

Along with the already mentioned direct interplay between autophagy and the SARS-CoV-2 proteins, the connection of this pathway with the receptor ACE2 can also interfere with viral replication. In the first place, the ubiquitination of ACE2 is recognized by the autophagic receptor TOLLIP that can deliver ACE2 for its lysosomal degradation by selective autophagy, reducing in turn, ACE2 availability and subsequent viral entry (105). Interestingly, the conjugation of the small ubiquitin-like modifier 3 (SUMO3) with ACE2 prevents its ubiquitination, and therefore, it enhances ACE2 stability and increases viral entry. Hence, the pharmacological inhibition of ACE2 SUMOylation has been suggested as a potential therapeutic strategy against SARS-CoV-2 (105).

On the other hand, ACE2 internalization may be directing SARS-CoV-2 virions post-endocytosis to the autophagy machinery through the direct interaction between the cytoplasmic C-terminal tail of ACE2 and the autophagy protein LC3. This interaction seems to be regulated by the phosphorylation of specific ACE2 motifs and therefore, it could also become a therapeutic target for the regulation of viral entry in epithelial cells (106).

## PHARMACOLOGICAL MODULATION OF AUTOPHAGY IN SARS-COV-2 INFECTION

As shown in Table 1, numerous studies have successfully restricted SARS-CoV-2 replication *in vitro*, *ex vivo,* and *in vivo* models by pharmacologically activating or repressing autophagy.

**TABLE 1:** Pharmacological treatments targeting autophagy during SARS-CoV-2 infection

Pharmacological treatments	Experimental model	Mechanisms of action	References
Rapamycin	*In vitro* and *in vivo*	Induces autophagy through the inhibition of mTORC1	80, 81
Spermine	*In vitro* and *ex vivo*	Induces autophagy through AMPK phosphorylation	81
Spermidine	*In vitro*	Induces autophagy through AMPK phosphorylation	81
SMIP004	*In vitro*	Induces autophagy by stabilizing BECN1	81
AR12	*In vitro*	Induces autophagosome formation	83
diABZI	*In vitro* and *in vivo*	Induces non-canonical autophagy via STING	84
Daurisoline	*In vitro*	Inhibits autophagy	107
Thapsigargin	*In vitro*	Inhibits autophagic flux	108
Chloroquine/hydroxychloroquine	*In vitro*	Inhibits lysosome fusion	109
ROC-325	*In vitro*	Inhibits lysosome fusion	109
Clomipramine	*In vitro*	Inhibits autophagic flux	109
Hycanthone	*In vitro*	Promotes lysosomal membrane permeabilization	109
Verteporfin	*In vitro*	Inhibits autophagosome formation	109
Mefloquine	*In vitro*	Inhibits autophagic flux	109
GNS561	*In vitro* and *in vivo*	Inhibits autophagic flux	110
3-MA	*In vitro* and *in vivo*	Inhibits autophagy via PI3K complex	80
SAR405	*In vitro*	Inhibits autophagy via VPS34 (PI3K)	80, 90
VPS34-IN1/VPS34-IN2	*In vitro*	Inhibits autophagy via VPS34 (PI3K)	80, 90

On the one hand, the induction of autophagy using different drugs, such as rapamycin, spermine, spermidine, or SMIP004, has shown a restrictive effect on SARS-CoV-2 replication and propagation in Vero cells, primary airways cells, and human organoids (81). In another study, the pharmacological activation of the autophagic flux using the compound AR12 successfully impaired viral replication in Vero cells in a dose-dependent manner (83). Interestingly, this inhibitory effect is not only limited to the canonical autophagic process. The induction of ULK1-independent autophagy using cyclic dinucleotides for the activation of STING1, had similar antiviral effect on SARS-CoV-2 (84).

On the other hand, and in agreement with the fact that SARS-CoV-2 manipulates autophagy to enhance its own replication, different studies have concluded that the inhibition of autophagy using compounds, such as Daurisoline, GNS561, 3-MA, Cyclosporine A, thapsigargin, or alisporivir, drastically impaired virus replication (80, 107 - 110). Interestingly, treatments with the autophagy inhibitor GNS561 not only caused an inhibition of the autophagic flux, which led to the accumulation of LC3-II vesicles (macroautophagic vesicles), but also enhanced the co-localization of SARS-CoV-2 with LAMP2 puncta and therefore, it could further support the potential role of CMA in the clearance of SARS-CoV-2 components (110). In addition to 3-MA, other inhibitors of the PI3K complexes, such as SAR405, VPS34-IN1, or VPS34-IN2, have also been proven to be very effective at repressing SARS-CoV-2 replication *in vitro* (80, 90). Overall, treatments with autophagy inhibitors successfully reduced virion production in different cell lines, primary human nasal, and bronchial epithelial cells and ameliorated virus-associated pneumonia using *in vivo* models as well as human lung tissues (80, 89).

## Summary and perspectives

Based on the current literature, the autophagy machinery seems to be acting as a double-edged sword in the context of SARS-CoV-2 infection. On the one hand, some studies point at the antiviral effect of autophagy and the concomitant counteraction by several viral proteins. On the contrary, many publications also describe how this virus is able, in various manners, not only to counteract such effect but also to induce and utilize the activation of the autophagy machinery to promote its own virion production and propagation. Hence, it is essential to conduct further research to comprehensively understand the complex relationship between SARS-CoV-2 and autophagy and thus, to be able develop effective therapeutic approaches against this virus. A deeper understanding of the potential implications of other types of autophagy, such as microautophagy or chaperone-mediated autophagy (CMA), could be beneficial in resolving some of the conflicting findings presented in the current literature.

Importantly, SARS-CoV-2-mediated manipulation of autophagy might also be accountable, at least partially, for some of the characteristic health problems associated with this COVID-19. In particular, autophagy dysregulation induced by SARS-CoV-2 has been linked to neuronal dysfunction in patients with COVID-19 (111). Furthermore, due to the role of autophagy in cytokine production, the modulation of autophagy has been proposed as potential measure to repress the damaging “cytokine storm” observed in COVID-19 patients (112). Therefore, it is imperative to increase our understanding about the interplay between SARS-CoV-2 and autophagy in order to find new therapeutic target and upgrade our capabilities to fight this virus, as well as to improve current COVID-19 patients’ conditions.
